# Unique β-Glucuronidase Locus in Gut Microbiomes of Crohn’s Disease Patients and Unaffected First-Degree Relatives

**DOI:** 10.1371/journal.pone.0148291

**Published:** 2016-01-29

**Authors:** Karine Gloux, Jamila Anba-Mondoloni

**Affiliations:** Micalis Institute, INRA, AgroParisTech, Université Paris-Saclay, 78350 Jouy-en-Josas, France; University of Illinois at Urbana-Champaign, UNITED STATES

## Abstract

Crohn’s disease, an incurable chronic inflammatory bowel disease, has been attributed to both genetic predisposition and environmental factors. A dysbiosis of the gut microbiota, observed in numerous patients but also in at least one hundred unaffected first-degree relatives, was proposed to have a causal role. Gut microbiota β-D-glucuronidases (EC 3.2.1.33) hydrolyse β-D-glucuronate from glucuronidated compounds. They include a GUS group, that is homologous to the *Escherichia coli* GusA, and a BG group, that is homologous to metagenomically identified H11G11 BG and has unidentified natural substrates. H11G11 BG is part of the functional core of the human gut microbiota whereas GusA, known to regenerate various toxic products, is variably found in human subjects. We investigated potential risk markers for Crohn’s disease using DNA-sequence-based exploration of the β-D-glucuronidase loci (GUS or Firmicute H11G11-BG and the respective co-encoded glucuronide transporters). Crohn’s disease-related microbiomes revealed a higher frequency of a C7D2 glucuronide transporter (12/13) compared to unrelated healthy subjects (8/32). This transporter was in synteny with the potential harmful GUS β-D-glucuronidase as only observed in a *Eubacterium eligens* plasmid. A conserved NH_2_-terminal sequence in the transporter (FGDFGND motif) was found in 83% of the disease-related subjects and only in 12% of controls. We propose a microbiota-pathology hypothesis in which the presence of this unique β-glucuronidase locus may contribute to an increase risk for Crohn’s disease.

## Introduction

Crohn's disease (CD) is a multifactorial incurable inflammatory bowel disease (IBD) of the human digestive tract whose etiology is unknown. It affects 26–200 per 100 000 persons in Europe [1]. It is thought that both genetic predisposition and environmental factors contribute to immune system problems. A positive family history is thought to be a predictive factor for 20% of IBD patients [2]. The number of independent human genetic loci reportedly contributing to CD easily exceeds 100, one third of which have been related to the innate immune system and autophagy pathways [3,4]. The genetic basis of CD is complex: genotyping alone is insufficient for prediction and does not explain what triggers remission and relapse. Increased frequency of CD in the industrialized countries is mainly explained by environmental risk factors [5] and a general bacterial dysbiosis is observed at the microbiome richness and bacterial species levels [6–11]. Studies of unaffected relatives have been recommended to resolve pathogenic mechanisms [12]. Two different microbiota dysbioses have been observed: one preceding CD and another inducing chronic CD-like ileitis [12–15]. No common marker has been clearly identified so that preventive measures can be taken.

β-glucuronidase (E.C.3.2.1.31) hydrolyses glucuronidated compounds, liberating glucuronic acid and the aglycone form that can be an imine, a thiol or an alcohol. It is co-encoded with a glucuronide transporter, allowing glucuronide entry in the bacteria and its use as carbon source. Among the thousands of species present in the human gut microbiota, a small number (around 50 species) carries genes encoding β-glucuronidases [16,17]. Two groups of glucuronidases are discerned based on amino-acid sequences [16,17], both representing relevant potential actors for a microbiota dysbiosis leading to disease. The GUS group is related to *E*. *coli* GusA and members are present in some strains of Firmicute, Actinobacteria and Proteobacteria [16]. The BG group, revealed by functional metagenomics, includes homologs to metagenomically identified H11G11 BG present in some strains of Firmicute and Bacteroidetes [16,17]. Numerous GUS substrates are naturally present in the diet or glucuronidated in the liver *via* the phase II detoxification pathway; endogenous metabolic wastes, vitamins, steroid hormones, animal- and plant-derived secondary metabolites, xenobiotics and pharmaceuticals are often conjugated with glucuronic acid [16,18–28]. GUS activity increases body exposure to the deglucuronidated form and is therefore efficient for exacerbating toxicity of hormones or drugs recognized by the human MRP1/MDR1 multidrug transporters or AhR aryl hydrocarbon receptor known to be crucial in IBD [29–32]. GUS β-glucuronidase is active on glucuronidated metabolites from nicotine [33] and notably, tobacco smoke is the only known environmental factor consistently predisposing to CD [5]. GUS β-glucuronidase activity is a prime etiology factor in the colon cancer [34,35] known to be more frequent in CD patients [36]. Furthermore the *gusAB* genes are present in the adherent-invasive *E*. *coli* implicated in the ethiopathogenesis of CD [37]. In contrast, β-glucuronidases of the BG group have unidentified natural substrates, but are part of the “healthy” functional core of the gut microbiota [17]. BG are present in Bacteroidetes and Firmicute, including Lachnospiraceae and Ruminococcaceae, two families that undergo population shifts in CD patients [2,9,38–40]. The Firmicute BG-positive strain *Ruminococcus gnavus* [17] is increased in CD patients and has been proposed as a CD signature [13]. Furthermore BG loci in Firmicute, and particularly co-localized transporters have been suggested to contribute to functional diversity and to adaptive mechanisms at the cell level [17]. Finally, despite the fact that dysbiosis in subpopulations potentially bearing beta-glucuronidases is observed in CD, beta-glucuronidase genetic loci have never been investigated in CD nor in unaffected first-degree relatives.

In the present study, we investigated β-glucuronidase loci (glucuronidase and co-localized transporter) as a potential factor of microbiota dysbiosis events preceding CD symptoms. We used sequence-based approaches to determine the relevance of GUS and Firmicute-BG loci as a mean to discriminate gut microbiomes of CD-related (CDR: patients and unaffected first-degree relatives) and CD-unrelated (CDU: CD healthy individuals).

## Materials and Methods

### Gut microbiota genomic databases

The CDR microbiomes explored in this study were from the Metahit project (http://www.metahit.eu/), including the rare available published database not restricted to 16S rDNA [41]. The CDR panel studied included four CD patients and eight unaffected first-degree relatives (Spanish individuals). The CDU healthy subjects included six Spanish individuals and twelve Danish individuals, randomly chosen from the Metahit project, and seven Japanese individuals from the ID 28117 project [42]. Major known information on the subjects used and metagenomic databases were recovered from publications from these two projects [41] [42] and presented in S1 Table. A total of thirty seven microbiomes were analyzed in this study.

### *In silico* analysis

Sequence-based exploration of CDR and CDU microbiomes for GUS and Firmicute-BG loci was performed using six genes as query sequences: β-glucuronidases and associated transporters from *E*. *coli* K12 (*gus*AB genes, NCBI accession numbers NP_416134 and NP_416133), and H11G11- and C7D2-BG and co-encoded transporters previously identified from a healthy subject and a CD patient, respectively [17] (metagenomic clones, EMBL accession numbers KC857626, KC857625 and FN666674, FN666673). Amino-acid sequences of the homologous H11G11 and C7D2 BGs have 44% identity, whereas those of the associated transporters have only 29% identity. Analyses of microbiomes were performed using tBlastn. Analyses using β-glucuronidases (GUS and H11G11) as queries did not reveal specific sequence signatures between human groups (supporting information). Amino acid sequence identities with both C7D2 and H11G11 transporters were determined after alignments with ClustalW. Percent identities were used to determine sequence convergence within each microbiome group (CD patients, CDR first-degree relatives and CDU healthy subjects) and their distribution was visualized using box-and-whiskers plot representation.

### Phylogenetic tree of H11G11 and C7D2 transporters from microbiomes and published genomes

We used H11G11 and C7D2 transporter sequences retrieved from microbiomes (≥30% identity) as queries to identify homologous genes from NCBI published sequences (genomes and nr/nt nucleotides collection). Alignments of H11G11, C7D2 transporters and the best homologs identified in microbiomes and genomes were performed using EXPRESSO multiple alignment tool of sequences and structures (3DCoffee::Regular, http://tcoffee.vital-it.ch/cgi-bin/Tcoffee/tcoffee_cgi/index.cg). A Neighbor-Joining tree was constructed using MEGA 5.5.

### Identification of a conserved motif in C7D2 transporters from CD related microbiomes

Alignment of NH_2_ terminal motifs (16 amino acids) was performed using ClustalW. A phylogenetic tree was performed using the Neighbor-Joining method. The pattern discovery tool PRATT (http://www.expasy.ch/tools/pratt/) was used to propose a conserved pattern for C7D2 transporters in CDR microbiomes.

### Analysis of conserved genes neighboring the C7D2 transporter

Gene neighborhoods of H11G11 and C7D2 transporters loci were determined from previously analyzed metagenomic inserts [17] and from sufficiently long sequences revealed by our *in silico* analysis of microbiomes. Gene prediction was performed using both Gene Mark tool (GeneMark.hmm for Prokaryotes, Version 2.8) and controlled with homologous predicted genes found in published genomes. Gene neighborhoods from published genomes were retrieved from the Doe Joint Genome Institute (http://img.jgi.doe.gov/cgi-bin/w/main.cgi).

### Statistical analysis

A Pearson's chi-squared test and a Mann-Whitney U-Test were performed to compare respectively male/female ratio and body mass index (BMI) between subjects bearing C7D2 transporter and other subjects. Mann-Whitney U-Tests were performed to compare variances of amino acid sequence convergence between CDR and CDU transporters or between Spanish CD, unaffected relatives and healthy subjects. The Fisher exact test was used to compare the frequency of C7D2 transporter in CDR and CDU subjects. Principal coordinate analyses (PCoA) ordination plots, based on Bray-Curtis distances were performed with identity percentages (≥30%) recovered from the cohort members using as queries: GUS and BG β-glucuronidases, C7D2 and H11G11 transporters as well as the C7D2 specific FGDFAND/ FGDFGND motif. PCoA results represent the distances through the identities with the queries used but not the real distances between all the amino acid sequences.

## Results

### The C7D2 transporter is more frequent in CDR than in CDU microbiomes

Using sequence-based exploration of microbiomes, we studied the potential use of β-D-glucuronidase loci sequences to discriminate between CDR (patients and unaffected first-degree relatives) and CDU (healthy subjects unrelated to CD). The exploration for both GUS and Firmicute-BG β-D-glucuronidase loci was performed using as queries: *E*. *coli* K12 GUS locus, H11G11 BG and C7D2 BG loci of two metagenomic clones from respectively a healthy subject and a CD patient [17]. No specific sequence signature was observed for GusA and BG and for the two transporters GusB and H11G11 (S1 and S2 Figs) but the C7D2 transporter revealed a good segregation between CDR and CDU subjects (S3 Fig). Two remarkable observations could be made: first, the C7D2 transporter was more frequent in CDR microbiomes than in CDU healthy subjects (based on >30% identity, S2 Table). It was present in 92% of CDR microbiomes, including unaffected relatives and in only 32% of CDU healthy subjects (p = 0.088). A CDR subject lacking the C7D2 transporter was eighteen years old and the youngest CDR subject, possibly suggesting a less advanced dysbiosis event. Statistical analyses were performed to determine a potential bias due to BMI and gender imbalances between the CDR and CDU groups. Among the four CD patients, one had a normal BMI and others a low weight as expected in CD. BMI were however not significantly different between the group bearing the C7D2 transporter and other subjects (p = 0.33), and high BMI were equally distributed in microbiomes with C7D2 transporters and H11G11 transporters. Gender distribution between the group bearing the C7D2 transporter and other subjects was different (p = 0.07). Women were however overrepresented among subjects bearing a C7D2 transporter but two subjects were men. Consequently neither BMI nor gender could be considered as a major bias in this study.

**Fig 1:**
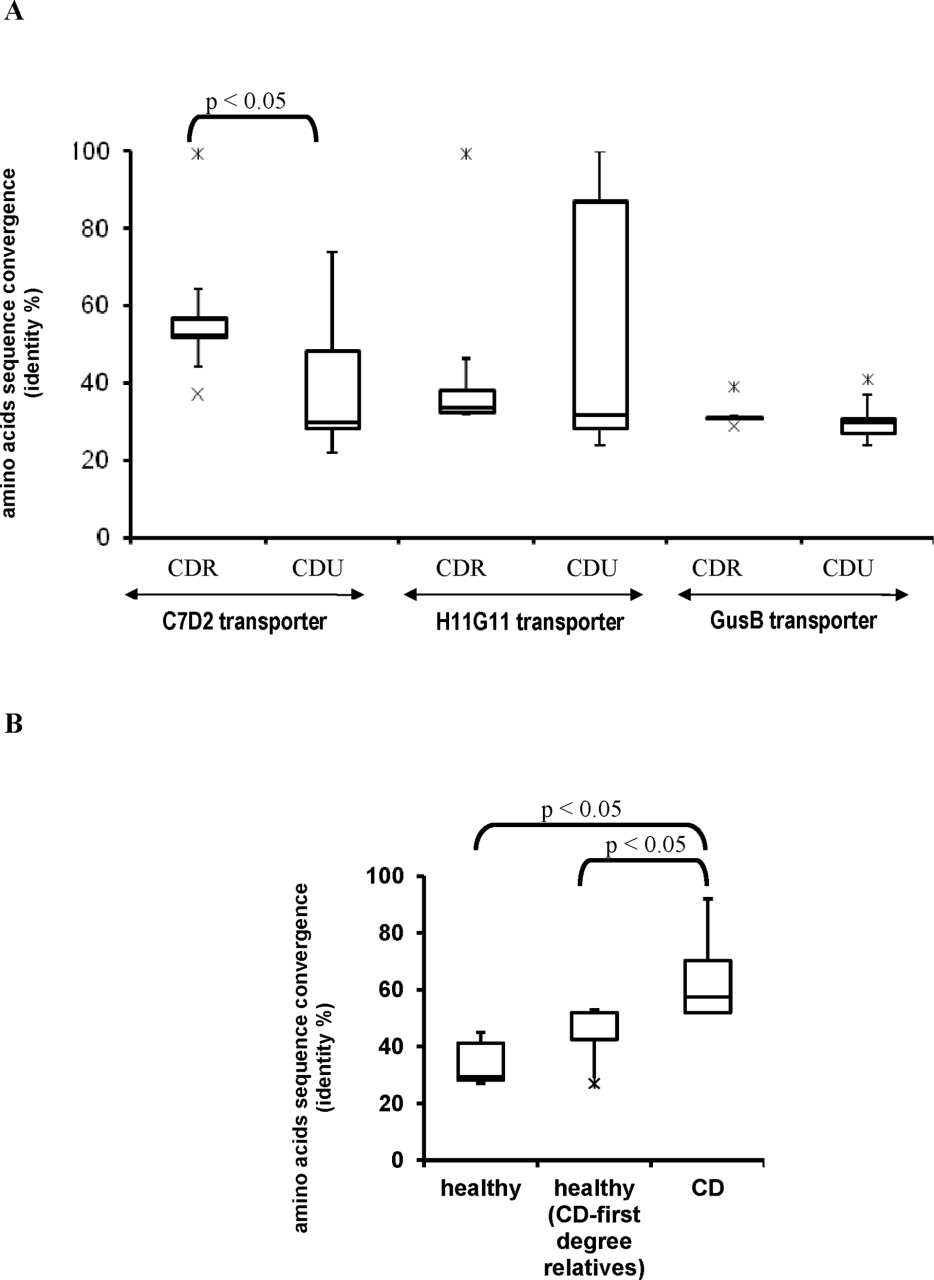
A conserved C7D2 transporter in microbiomes from CD patients and unaffected first-degree relatives (CDR). **(A)** Boxplot representation of percent identities between C7D2, H11G11 or GusB transporters and homologs from CDR and CDU microbiomes. **(B)** Analysis restricted to Spanish subjects and homologs of C7D2 transporter. Homologs of H11G11 and C7D2 transporters were searched, using the tBlastn tool, in human gut microbiomes from sick and healthy first-degree relatives CD related (together referred to as CDR) and CD unrelated healthy subjects. Representation was performed per query (C7D2, H11G11 and GusB transporters) for each disease status. Results are presented as percent amino acid identity between each query and homologs recovered from microbiome sequences. Comparisons of variance between amino acid sequence convergence of CDR and CDU transporters or between Spanish CD, unaffected relatives and healthy subjects were performed using a Mann-Whitney U-Test.

The second main observation was that C7D2 transporters from CDR microbiomes had high amino acid sequence conservation not observed for H11G11 or GusB transporters (Fig 1A, S2 and S3 Figs). Even if a larger panel will be necessary to discriminate between patients and asymptomatic CDR, we noticed that Spanish CD microbiomes had significant higher conserved sequences of C7D2 transporters than both CDU and asymptomatic CDR Spanish subjects (Fig 1B and S3 Fig). This *in silico* analysis based on β-D-glucuronidase loci indicated that most CDR subjects had a gut microbiome equipped with a conserved C7D2 transporter.

### The C7D2 transporter is plasmid-encoded or present on specific Clostridiale chromosomes

**Fig 2:**
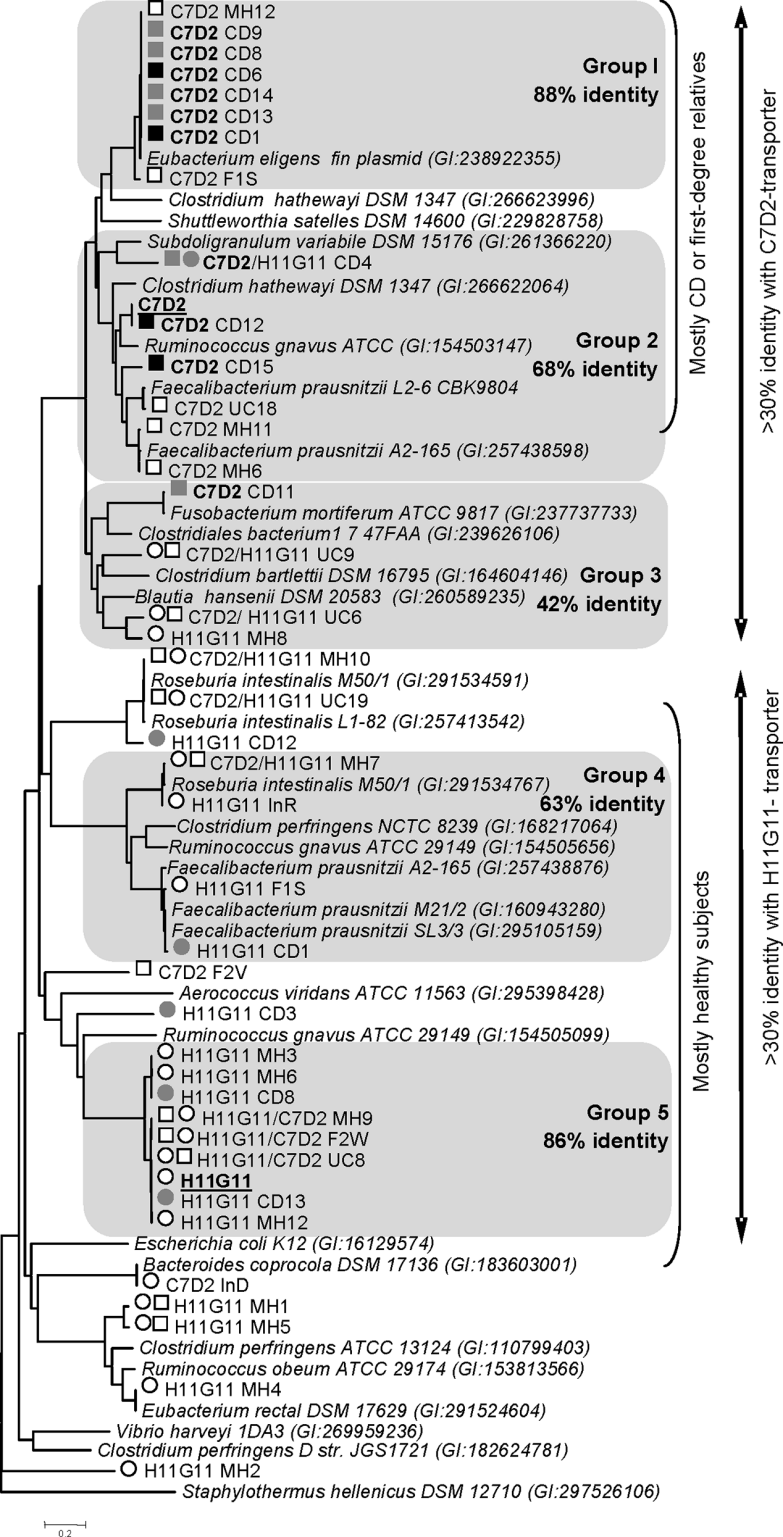
Phylogenetic tree of H11G11 and C7D2 transporters in CDR and CDU microbiomes. Analysis was performed as described in Fig 1. Twenty nine protein sequences were retained (>30% identity, > 80% coverage) from the best Blast results for each query. The phylogenetic assignments were performed by searching homologs in published genomes (see S3 Table). All protein sequences were aligned using Expresso (3D Coffee) and a Neighbor-Joining tree was performed using MEGA 5.5. Groups are proposed for clusters within high identity percentages (>42% within each group). Sequence names are referred to as: query used/ microbiome sequence name (Metahit and ID 28117 projects). Black and white symbols are used to distinguish CD-related subjects (patients and asymptomatic first-degree relatives; CDR) and CD-unrelated healthy subjects, respectively. The queries used (H11G11 and C7D2 transporters) are underlined. Squares and circles correspond to results using respectively H11G11 and C7D2 transporters as query. This *in silico* exploration of CDR and CDU microbiomes reveals that H11G11 transporter is mostly present in healthy subjects, whereas C7D2 transporter is recovered in CDR subjects, including unaffected relatives. The C7D2 transporter is plasmid-encoded or present on specific Clostridiale chromosomes.

The C7D2 transporters from the CDR microbiomes grouped in a clade (>42% identity) and, as expected, co-clustered with sequences from published Firmicute genomes (identity score ≥ 50%, Fig 2 and S2 Table). Half of them were surprisingly clearly assigned (> 98% identity) to an unnamed plasmid (NC-012780) issued from *Eubacterium eligens* ATCC 27750. Other C7D2 transporters recovered from microbiomes constituted a group with high sequence conservation (>66% identity), which were homologous to those found in *Subdoligranulum variabile* DSM 15176, *R*. *gnavus* ATCC 29149, *Clostridium hathewayi* DSM 13479, and specific strains of *Faecalibacterium prausnitzii* (L2/6 and A2-165). Interestingly three of these strains, *R*. *gnavus*, *F*. *prausnitzii* A2-165 and *C*. *hathewayi* DSM 13479, were equipped with both C7D2 and H11G11 transporter types (2 to 4 copies per genome). In conclusion, these results showed that the C7D2 transporters may be plasmid- or chromosome-carried in a few Clostridiale, well equipped for β-glucuronidase-linked functional diversity.

### Genes encoding C7D2 transporters from CDR microbiomes co-localize with *gusA* genes

**Fig 3:**
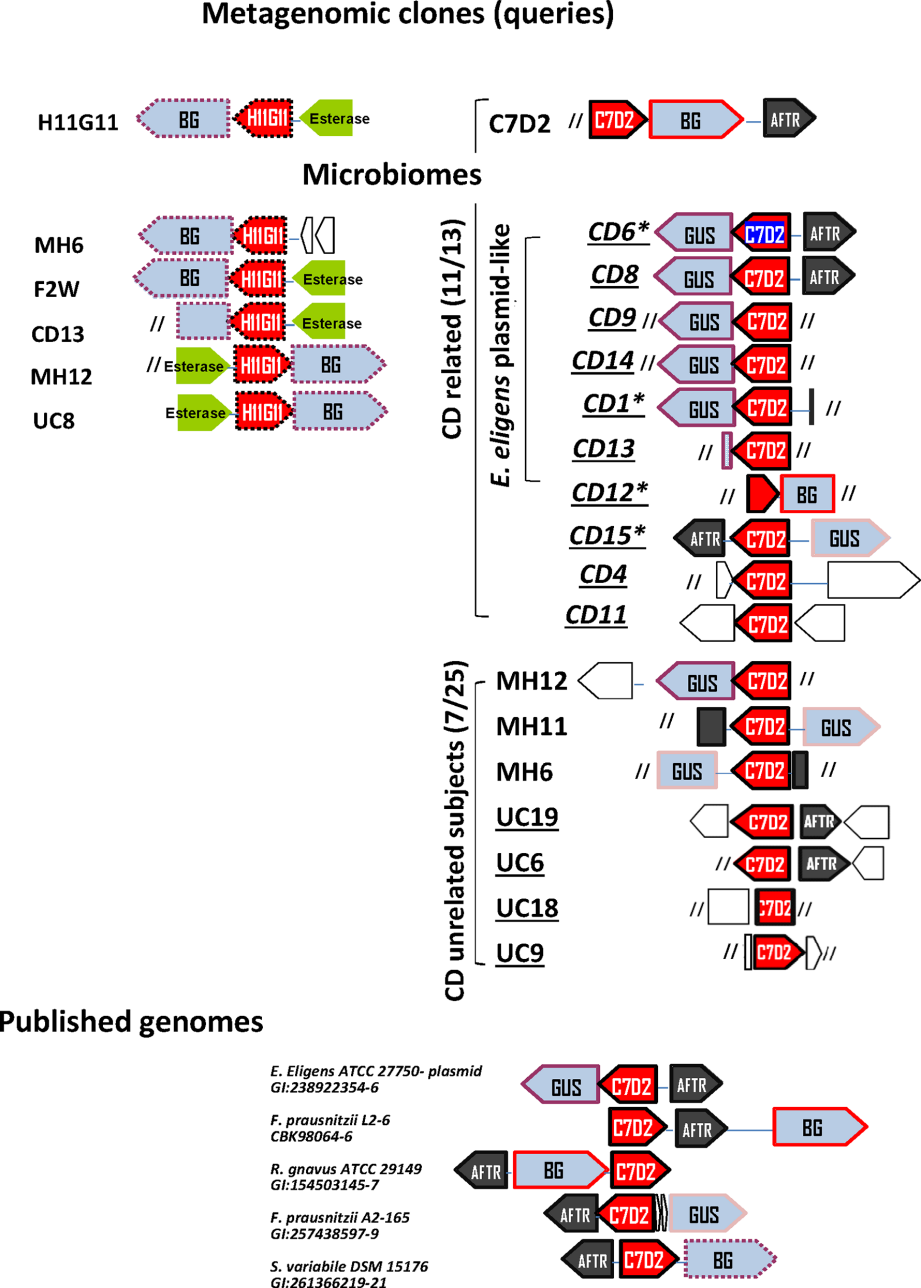
Gene neighborhood views of H11G1 and C7D2 transporters loci. We analyzed genes that co-localized with C7D2 or H11G11 transporters from groups 1, 2 and 5 as previously defined (Fig 2). Gene neighborhood views from microbiomes were obtained using the Gene Mark prediction tool and annotated published genomes. Red box, glucuronide transporter; blue box, BG or GUS β-glucuronidases; black box, AraC family transcriptional regulator; brown box, putative esterase lipase; empty box, putative protein; slashes, sequence end. Homologous sequences are surrounded by same color lines. *: CD patient. Spanish subjects are underlined. This analysis of genes neighboring H11G11 and C7D2 transporters reveals that most CDR have an unexpected C7D2 transporter/GUS β-glucuronidase locus.

C7D2 transporters in CDR microbiomes were in keeping with glucuronide transport function; most of them co-localized with a β-glucuronidase gene and were within a single transcriptional unit including *gusA*, as shown for *E*. *eligens* plasmid (http://biocyc.org/gene?orgid=EELI515620&id=GH1N-2656#tab=TU/). The C7D2 transporter was first identified in a Firmicute BG locus from a metagenomic insert (*E*. *coli* as the receiving bacteria) and from several published genomes [17]. Here we noted that C7D2 transporters from most CDR microbiomes unexpectedly co-localized with a GUS β-glucuronidase (100% identity with PS00719 and PS00608 Prosite consensus GUS signatures) (Fig 3 and S4 Fig). The GUS protein from the *E*. *eligens* plasmid had 45% identity with *E*. *coli* GusA known for its harmful activity [22,23].

The second observation was the co-localized occurrence of an AraC family transcriptional regulator (AFTR) (Fig 3) characterized by a conserved DNA-binding domain (PS01124 Prosite pattern, S5 Fig). AFTR is a master virulence regulator that responds to chemical signals [43,44]. Two observations argue for a functional link between AFTR and the C7D2 transporter: i) AFTR is grouped like respective co-localized C7D2 transporters (based on their phylogenetic tree, S6 Fig) and ii) published genomes with assigned C7D2 transporters were those with a neighboring full length AFTR (>84% coverage, S3 Table). Finally the GUS/C7D2 transporter/AFTR locus was present in 50 to 75% of CDR microbiomes and in only 8 to 16% of CDU microbiomes (taking into account interrupted sequences). According to known GUS activities and inflammatory properties of potential substrates, this locus likely reflects a high potentiality for a harmful inflammatory response.

### N-terminal motif of the C7D2 transporter: a candidate marker for CD predisposition

**Fig 4:**
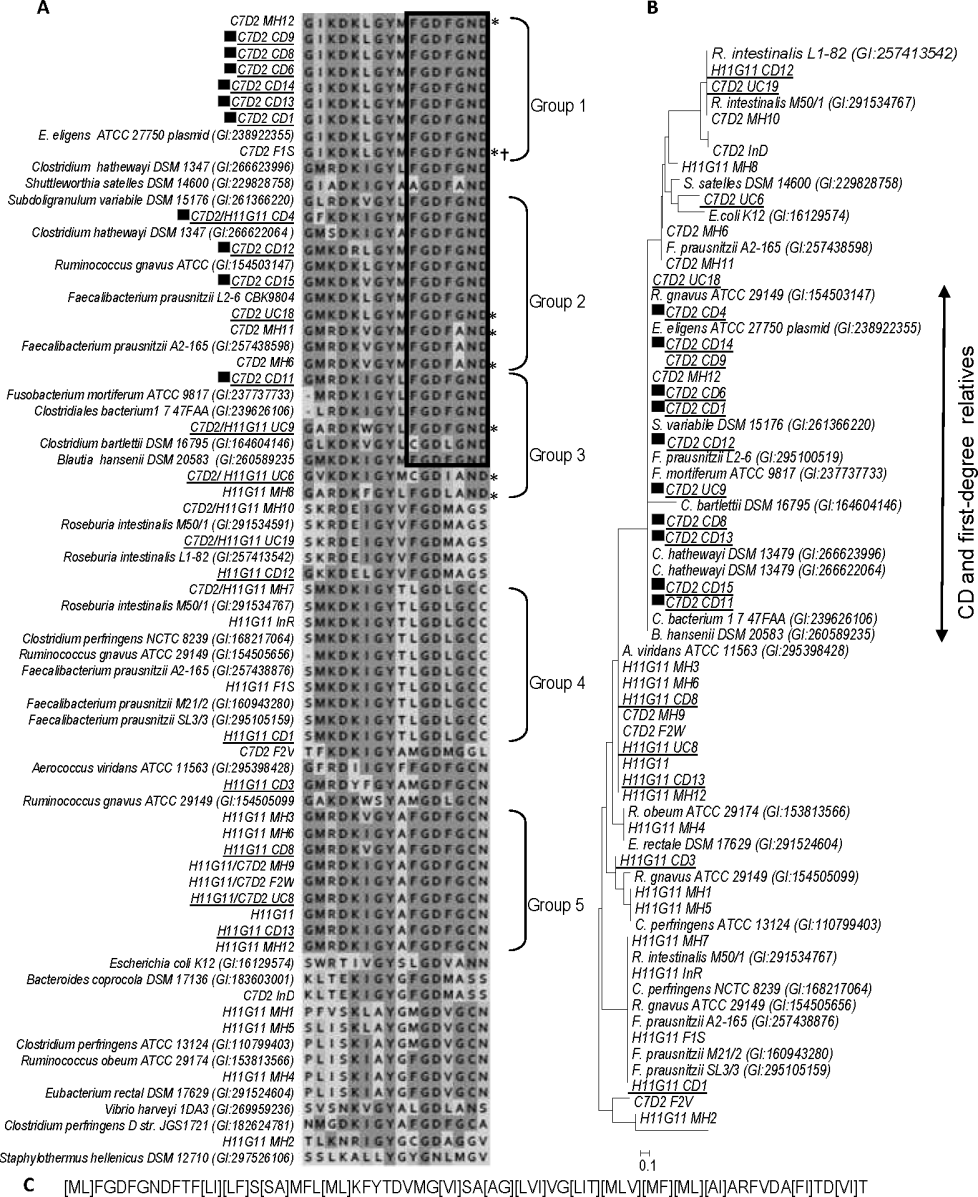
A conserved NH2-terminal motif in C7D2 transporters from CD related microbiomes. **(A)** Alignment of NH2-terminal motifs from H11G11 and C7D2 transporters. NH2 terminal motifs were extracted from Expresso (3D Coffee) alignment performed from H11G11 and C7D2 transporters sequences presented Fig 1. * CD unrelated healthy subjects with C7D2 transporter, † no ORF predicted. Black squares indicate C7D2 transporter from CD related microbiomes. Spanish subjects are underlined. **(B)** Phylogenetic tree based on an alignment restricted to the “FGDFGND” NH2-terminal motif. Neighbor-Joining method was used to perform the tree. Sequences from F1S and C7D2 are absent because the first has no predicted ORF and the second has a truncated NH2-terminal sequence. **(C)** Proposed pattern for a beta-glucuronidase linked transporter specific of CD related microbiomes. The conserved pattern was determined using C7D2 transporter sequences from CD related microbiomes and published genomes (carrying GND-end motif) as found by the pattern discovery tool PRATT (http://www.expasy.ch/tools/pratt/).

As conserved C7D2 transporter was shown to discriminate CDR from most CDU subjects, the conserved regions were analyzed in more detail. The longest conserved sequence was a FGDFGND N-terminal motif (S7 Fig) present in all C7D2 transporters recovered from CDR subjects and in rare CDU healthy subjects (3/25, Fig 4A and S8 Fig). According to a phylogenetic tree from an alignment of the FGDFGND motif, 83% (10/12) of CDR members are in a same cluster and 88% of the CDU healthy subjects were excluded (Fig 4B). Moreover a conserved alanine to glycine substitution (FGDFAND) was observed in 4/7 C7D2 transporters (>30% identity) found in CDU microbiomes from both Spanish and Danish origin (Fig 4A and S8 Fig). Consequently none of the CDU healthy subjects had the GUS/FGDFGND C7D2 transporter/AFTR association. The strict FGDFGND motif was also identified in most C7D2 transporter homologs from published genomes: *E*. *eligens* (plasmid-carried), *S*. *variabile* DSM 15176, *R*. *gnavus* ATCC 29149, *C*. *hathewayi* DSM 13479, and *F*. *prausnitzii* L2-6. Interestingly, these bacteria belong to families (i.e., Lachnospiraceae, Ruminococcaceae) that undergo population shifts in CD patients. The two *F*. *prausnitzii* strains carrying a C7D2 transporter (A2-165 and L2-6) belong to the same 16S rRNA phylogroup. However, they were differentiated by both the [G/A] substitution and their capacity to grow on glucuronic acid [45], raising the question of a potential role of GUS/FGDFGND in transporter functionality or its regulation. Finally, the GUS/FGDFGND C7D2 transporter/AFTR association found in the *E*. *eligens* plasmid was present only in CDR microbiomes.

Based on our analyses, we propose a pattern for a glucuronide transporter associated to CD related status (Fig 4C) and the FGDFGND motif as a predictive tool of a microbiota dysbiosis prior to the onset of CD.

## Discussion

The bacterial β-glucuronidases of the gut microbiota include harmful GUS enzymes involved in the retention of toxic compounds, and BG enzymes that are part of the ‘healthy’ functional core [16–28]. Several previous studies, including unaffected CD relatives, supported a microbiota predisposition hypothesis [12–14] but a functional explanation was missing. The aim of this study was to investigate the potential shift in β-glucuronidase loci of the gut microbiota in patients and unaffected relatives. A screen of microbiomes for GUS and Firmicute-BG loci was performed on available databases from CDR subjects and compared to CDU healthy subjects. The present β-glucuronidase-based approach proposes a bio-marker of bacterial dysbiosis predisposing to CD. In this study, a C7D2 transporter is shown as particularly conserved in published CDR microbiomes and unexpectedly associated to a GUS β-glucuronidase. More specifically, a FGDFGND motif of C7D2 transporters discriminates CDR from CDU microbiomes. A FGDFAND point substitution, consistent with a difference in the ability to use glucuronic acid as a carbon source [45], is found in homologs from CDU microbiomes. These results lead us to propose a functional hypothesis of a β-glucuronidase contribution to microbiota dysbiosis predisposing to CD.

Markers of CD predisposition were extensively explored in human genomes; at least 140 risk loci were proposed [1]. The best markers are localized in the NOD2 locus and are more present in CD patients (30–40%), but are also present in healthy subjects (6–7%) [1]. The gut microbiota dysbiosis is currently considered as a marker valuable in identifying individuals at risk, but the diagnostic is time consuming and expensive. Our data do not offer a mechanistic role for this motif in CD predisposition, but propose the NH2-terminal sequence as a marker found in 83% of CDR, compared to only 12% of CDU (i.e., which is more discriminating than NOD2 as a marker). Similarly, the GUS/C7D2 transporter/AFTR locus was estimated as present in 50–75% of CDR against only 8–16% CDU.

The C7D2 transporter is most readily attributable to a plasmid in *E*. *eligens*, followed by *R*. *gnavus* and *F*. *prausnitzii* genomes. These bacteria are part of the common set of fecal microbiota species and belong to *Clostridium* clusters IV and XIVa [41,46]. It is tempting to speculate that horizontal gene transfer from the *E*. *eligens* plasmid might be implicated in events predisposing to CD. Gut inflammation reportedly stimulates horizontal gene transfer and shifts the current paradigm of the “separate” evolution of pathogens and commensals [47]. Horizontal *gus* gene transfer has been identified in bacteria [48,49] and more particularly is suggested in *F*. *prausnitzii* A2-165 [16], identified in the present work as the only bacterial genome bearing a C7D2 transporter/GUS locus. CDR microbiomes may be exposed to excessive glucuronide sources, thus generating a gut microbiota activated for lateral transfer of *gus*-related genes. Only the *E*. *eligens* plasmid bears the strict GUS/FGDFGND C7D2 transporter/AFTR locus found in CDR microbiomes. Even if this organization was not described in other published genomes, *F*. *prausnitzii*, *R*. *gnavus* or *S*. *variabile* species can be proposed as other actors in shifts specifically induced in CDR subjects.

What is the potential functional meaning for the presence of this locus in both CD patients and in first-degree relatives? All CDR subjects do not develop symptoms, and the presence of the GUS/FGDFGND C7D2 transporter/AFTR locus cannot be systematically associated to development of Crohn’s disease. However, according to known GUS activities, this locus can be involved in the liberation of pro-inflammatory products from environmental factors. Indeed, the C7D2 transporter/GUS loci, mostly assigned to the *E*. *eligens* plasmid, are likely harmful microbiota factors in CDR subjects since both the GusA activity and *E*. *eligens* ATCC 27750 have been demonstrated to activate mutagenic compounds [22,50]. Several known GusA substrates coincide with both endogenous and environmental factors implicated in CD: nicotine [33] from tobacco smoke, the only environmental factor consistently predisposing to CD [5], and hormones or drugs [27,28] recognized by host transporters (MRP1/MDR1) or receptors (AhR) that are crucial in IBD [29–32]. It is likely that MDR1 polymorphisms, associated with a predisposition to CD [51], contribute to inter-individual variation of susceptibility to compounds reactivated by GusA activity. Interestingly, the unaffected CD first-degree relative subjects used in this study had high BMI, known to be associated to inflammation and a dysbiosed microbiota [52]. This observation is in accordance with an inflammatory status predisposing to CD and raises the controversial question of whether being overweight is an initial step before appearance of CD disease [53–55].

*F*. *prausnitzii* and *R*. *gnavus*, recurrently highlighted in CD microbiota dysbiosis [2,39,56–59], are among the five species proposed as characterizing the disease [13] and *Subdoligranula* genera has been also considered as a CD dysbiosis marker [2]. The FGDFAND point substitution discriminates *F*. *prausnitzii* strains in accordance with ability/inability to use glucuronic acid as a carbon source [45]. It was recovered from healthy subjects in sequences assigned to *F*. *prausnitzii* known to have anti inflammatory property and to be decreased in CD [2,58–60]. In contrast *R*. *gnavus*, known to be increased in CDR versus CDU subjects [13,57], bears the FGDFGND C7D2 transporter found in affected CD patients. These results may suggest that the transporter has a different function when present in CDR *versus* CDU subjects. Further investigation is necessary to determine the complex mechanisms that differentiate *R*. *gnavus* and *F*. *prausnitzii* dysbiosis.

If as observed by Joossens et al [13], the species implicated in dysbiosis of microbiota from CD patients are not the same as in unaffected relatives, the microbiota functional events preceding symptoms likely differ from those after the first CD symptoms. It is thus unlikely that the GUS locus is a single disease-promoting factor, nor can it be unequivocally linked to CD severity. However its presence provides support for an increased susceptibility to pro-inflammatory compounds contributing to predisposition. The difference in species dysbiosis previously observed between unaffected CDR and patients [13] argues for a strong resilience of strains equipped with this locus.

**Fig 5:**
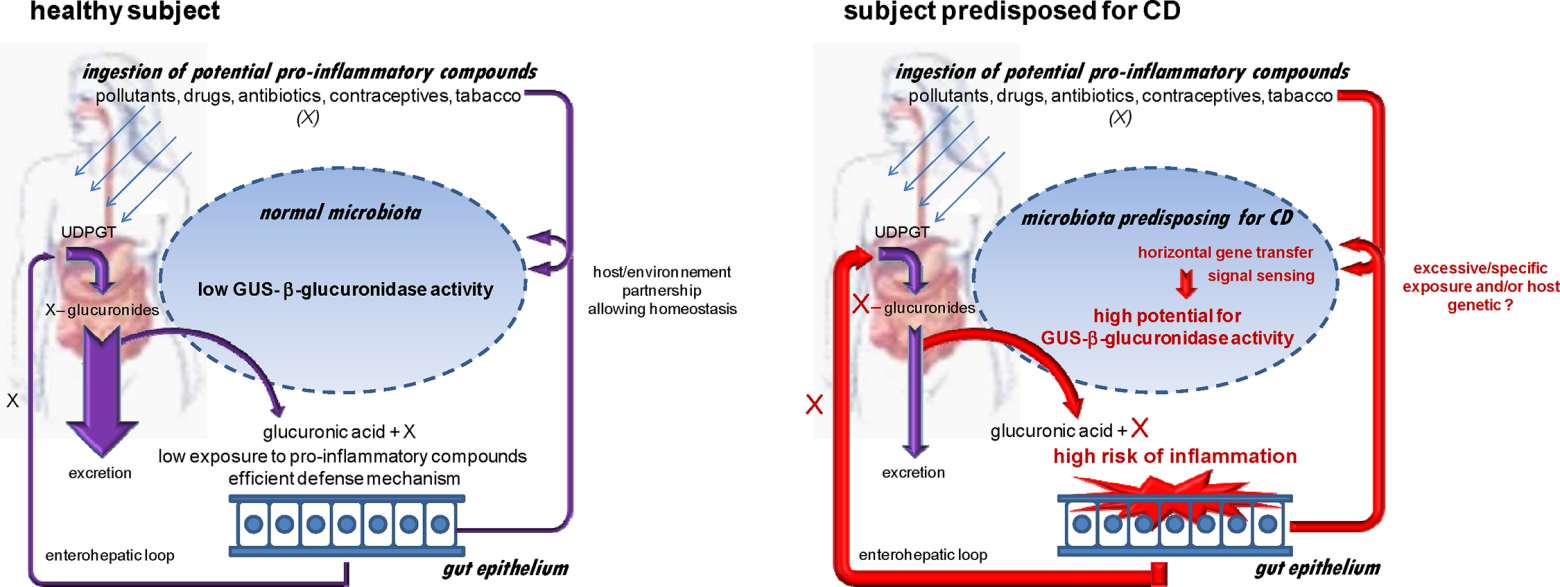
Pro-inflammatory microbiota hypothesis involving the unexpected GUS/C7D2 transporter/AFTR locus and predisposing to CD. Diverse pro-inflammatory compounds ingested in the human body are detoxified to more water-soluble forms via the liver phase II pathway that include glucuronidation by glucuronosyl-transferases (UDPGT). The glucuronidated compounds are mostly excreted in bile and finally eliminated in faeces. The GUS activity of the gut microbiota releases the lipid-soluble/pro-inflammatory form, able to reenter the enterohepatic loop and therefore elevate risk of inflammation and body exposure. In healthy subjects, despite individual specificities, the GUS equipment remains compatible with homeostasis. In subjects predisposed to CD, a higher potential for GUS activity is hypothesized to result from expression of the GUS/FGDFGND C7D2 transporter/AFTR locus. Even if all unaffected CDR do not develop CD, this potential GUS-linked damage precedes symptoms and may be involved in exacerbation of chronic inflammation in response to environmental and/or host genetic signals.

We propose a pro-inflammatory microbiota hypothesis involving the unexpected GUS/FGDFGND C7D2 transporter/AFTR locus (Fig 5). In a CDU healthy subject, bacterial β-glucuronidase activities are compatible with microbiota homeostasis. In both CD patients and asymptomatic predisposed subjects, environmental and/or genetic factors lead to emergence of GUS/C7D2 transporter/AFTR loci with increased potential to release pro-inflammatory products. Diverse etiological factors suspected in CD, such as smoking, diet, pollutants, antibiotics or oral contraceptives [5] are sources of glucuronides [61–63], and our data may point to a physiological convergence linked to bacterial GUS loci activities.

In conclusion, the C7D2 transporter (characterized by the FGDFGND motif) adjacent to a *gus* gene is proposed as a locus associated to microbiota dysbiosis observed in both patients and unaffected relatives. Further investigation of individuals from different countries and ethnic groups to identify potential relevant sequence variation may uncover a universal diagnostic tool for predisposition. AFTR, a target for new strategies to prevent or treat bacterial infections [43] can be proposed to prevent CD in at-risk individuals. A long term follow-up of this locus in unaffected first-degree relatives that develop or not the disease may give further insight into what tools are most useful for prevention.

## Supporting Information

S1 Fig β-glucuronidase dispersion in CDR and CDU. **(a)** GUSA protein. **(b)** H11G11 BG protein. Principal coordinate analyses (PCoA) ordination plots, based on Bray-Curtis distances were performed with identity percentages recovered from the cohort members using *E*. *coli* K12 GUSA and metagenomic H11G11 BG as query. *: truncated protein, blue: CDU subjects, black: CDR asymptomatic subjects, bold black: CD patients.
(PDF)

S2 Fig H11G11 and GUSB transporters dispersion in CDR and CDU. Principal coordinate analyses (PCoA) ordination plots, based on Bray-Curtis distances were performed with identity percentages recovered from the cohort members using metagenomic H11G11 transporter as query. *: truncated protein, blue: CDU subjects, black: CDR asymptomatic subjects, bold black: CD patients.
(PDF)

S3 Fig C7D2 transporter dispersion in CDR and CDU. Principal coordinate analyses (PCoA) ordination plots, based on Bray-Curtis distances were performed with identity percentages recovered from the cohort members using metagenomic C7D2 transporter as query. **(a)** All CDR and CDU tested. **(b)** Only Spanish subjects. *: truncated protein, blue: CDU subjects, black: CDR asymptomatic subjects, bold black: CD patients.
(PDF)

S4 Fig Putative β-glucuronidases associated to C7D2 and H11G11 transporters. The Neighbor-Joining tree was performed using MEGA 5.5 on β-glucuronidase sequences close to C7D2-and H11G11- transporters from microbiomes and to C7D2-transporters from published genomes. Red squares and circles represent respectively GUS and BG β-glucuronidases neighboring a C7D2-transporter. Δ metagenomic clone from CD patient (*E*. *coli* as the host), * microbiome from CDR including patients and unaffected first degree relatives, ° microbiome from healthy subject.
(PDF)

S5 Fig Protein alignments of AraC family proteins from C7D2-transporter loci and homologs from published genomes. The AraC-like sequences retrieved from C7D2 transporter loci (mostly from CDR microbiomes) and published genomes were aligned using the Tcoffee expresso tool (TCoffee score 96–98). The COOH-terminal domain encodes the DNA binding domain.
(PDF)

S6 Fig Phylogenetic tree of putative transcriptional regulators neighboring C7D2 transporters. Neighbor-Joining tree was performed using BLOSUM62 on region after ClustalW alignment from AFTR sequences co-localized with groups 1 and 2 C7D2-transporters (see Fig 1) and those of the best homologs (YSIRK-targeted surface antigen AFTR) found in published genomes. *: CDR, °: healthy subject. AFTRs grouped like their respective co-localized C7D2 transporters.
(PDF)

S7 Fig Sequence alignment of C7D2 transporters from CD related microbiomes. The Alignment was performed using Expresso multiple alignment tool of protein sequences using structural information (T-COFFEE Multiple Sequence Alignment Server).
(PDF)

S8 Fig FGDFGND motif dispersion in CDR and CDU. Principal coordinate analyses (PCoA) ordination plots, based on Bray-Curtis distances were performed with identity percentages recovered from the cohort members using the FGDFGND motif from the metagenomic C7D2 transporter as query. CDU subjects, black: CDR asymptomatic subjects, bold black: CD patients.
(PDF)

S1 Table Summary of the samples and sequencing. (PDF)

S2 Table Best tBlastn results for H11G11- and C7D2- transporters in microbiomes of CD patients, first degree relatives and healthy subjects. (PDF)

S3 Table Coverage and identity of amino acid AFTR sequences associated to C7D2-transporter in published genomes. (PDF)
