# Supplementary figures and images for: Unique β-Glucuronidase Locus in Gut Microbiomes of Crohn’s Disease Patients and Unaffected First-Degree Relatives
Source: PLoS One. 2016 Jan 29;11(1):e0148291. doi: 10.1371/journal.pone.0148291 (PMC4732671; doi:10.1371/journal.pone.0148291)

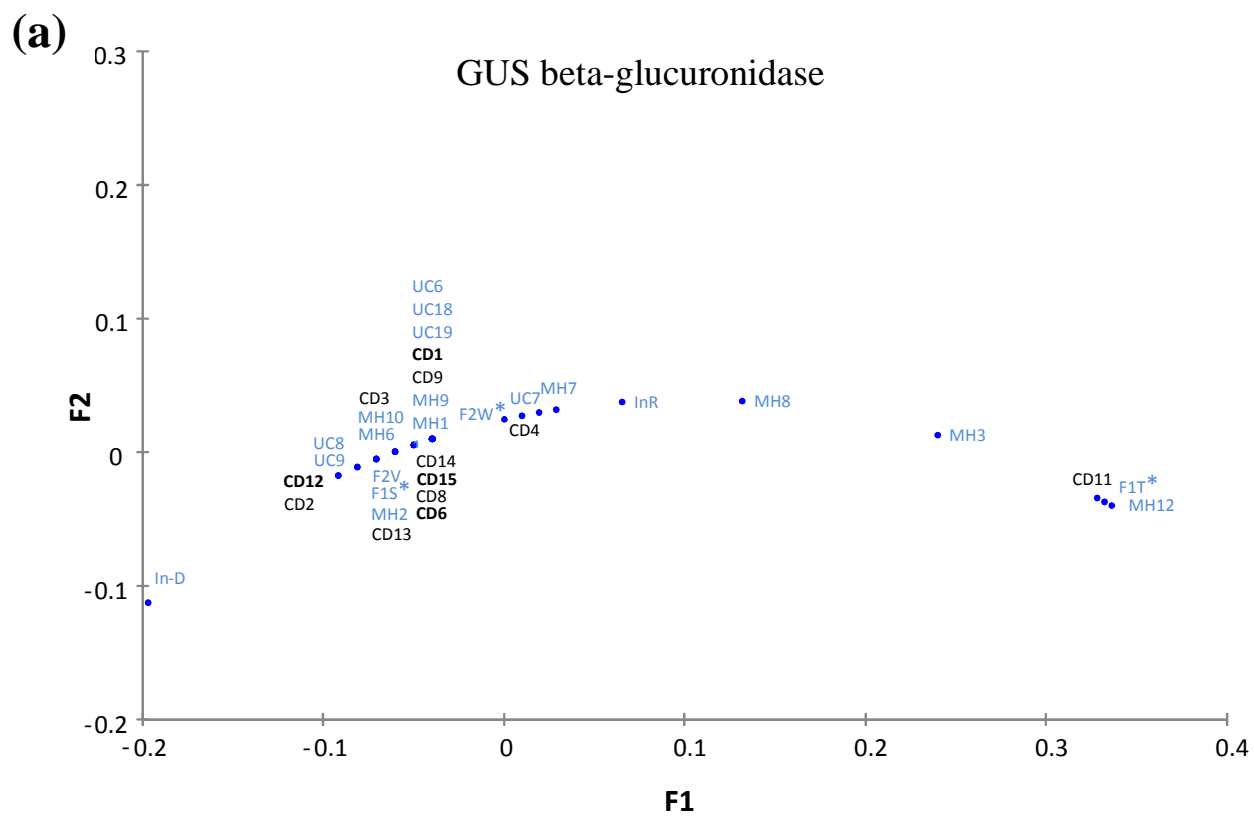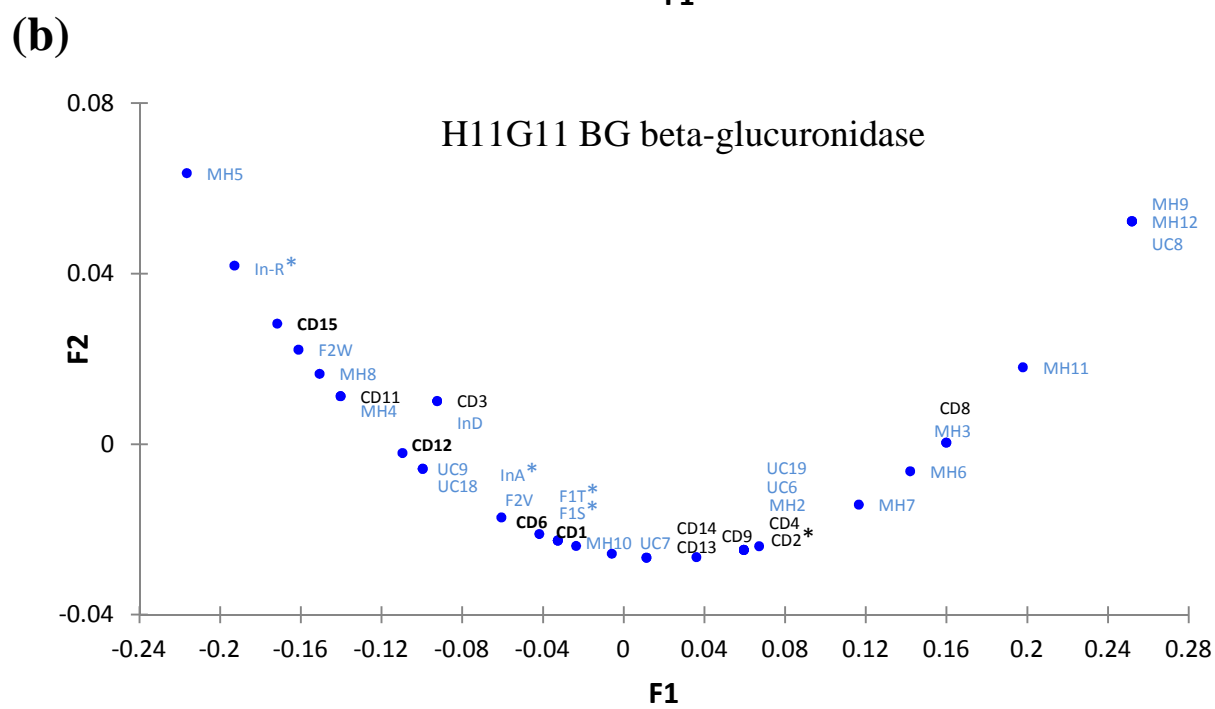

**S1 Figure**

Supplement: S1 Fig — (a) GUSA protein. (b) H11G11 BG protein. Principal coordinate analyses (PCoA) ordination plots, based on Bray-Curtis distances were performed with identity percentages recovered from the cohort members using E. coli K12 GUSA and metagenomic H11G11 BG as query. *: truncated protein, blue: CDU subjects, black: CDR asymptomatic subjects, bold black: CD patients. (PDF) [file pone.0148291.s001.pdf]

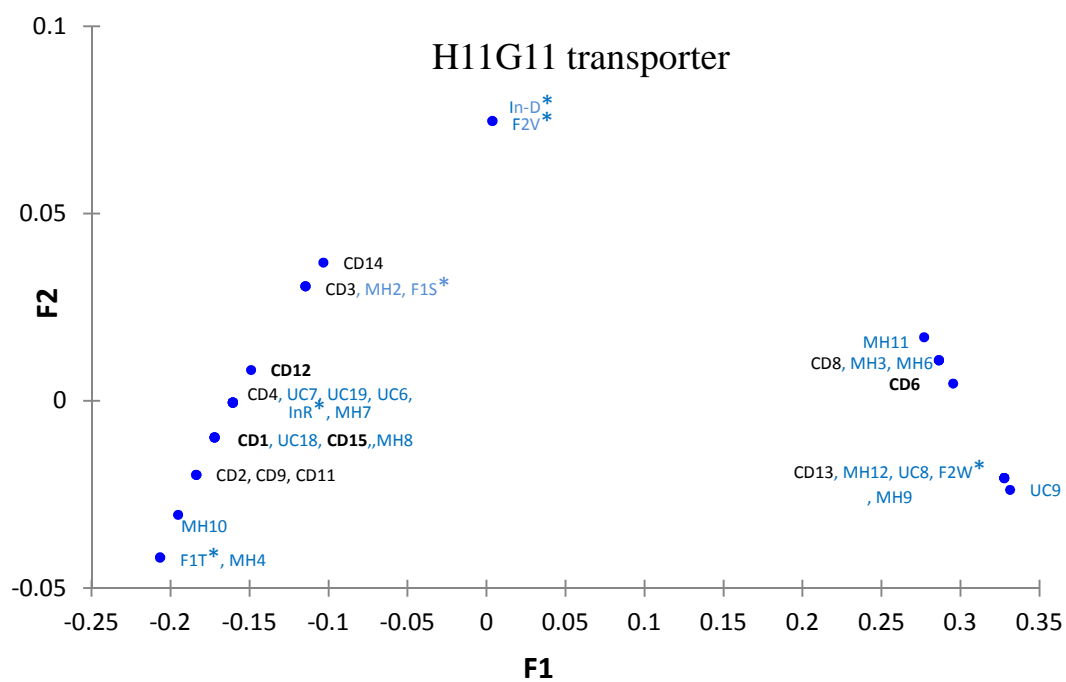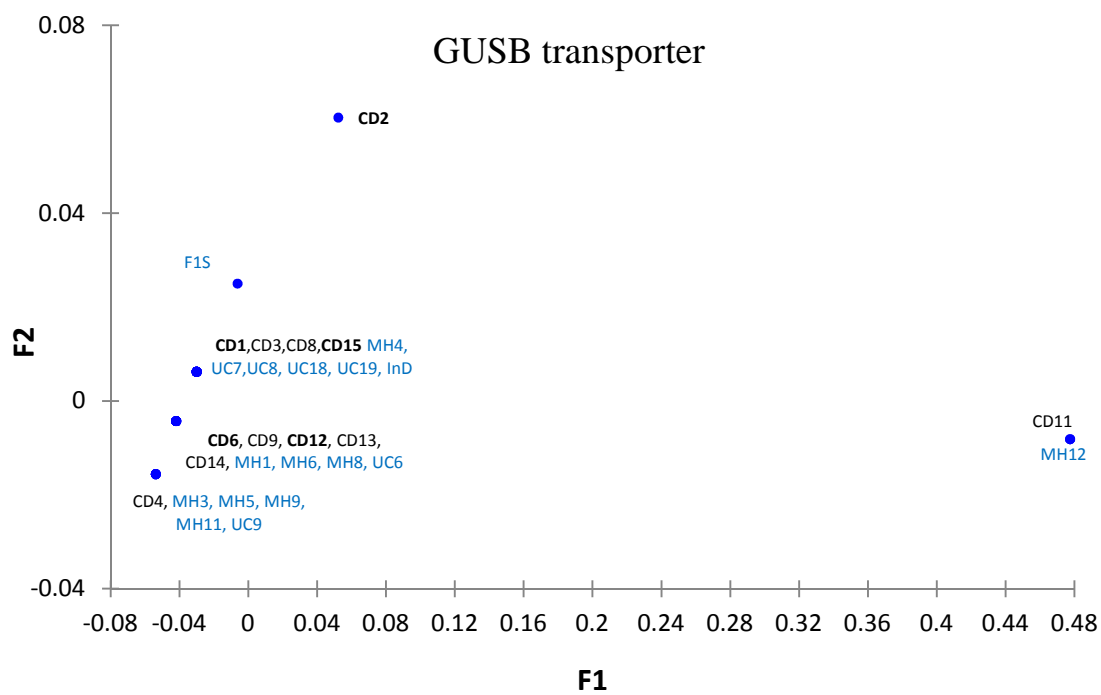

**S2 Figure**

Supplement: S2 Fig — Principal coordinate analyses (PCoA) ordination plots, based on Bray-Curtis distances were performed with identity percentages recovered from the cohort members using metagenomic H11G11 transporter as query. *: truncated protein, blue: CDU subjects, black: CDR asymptomatic subjects, bold black: CD patients. (PDF) [file pone.0148291.s002.pdf]

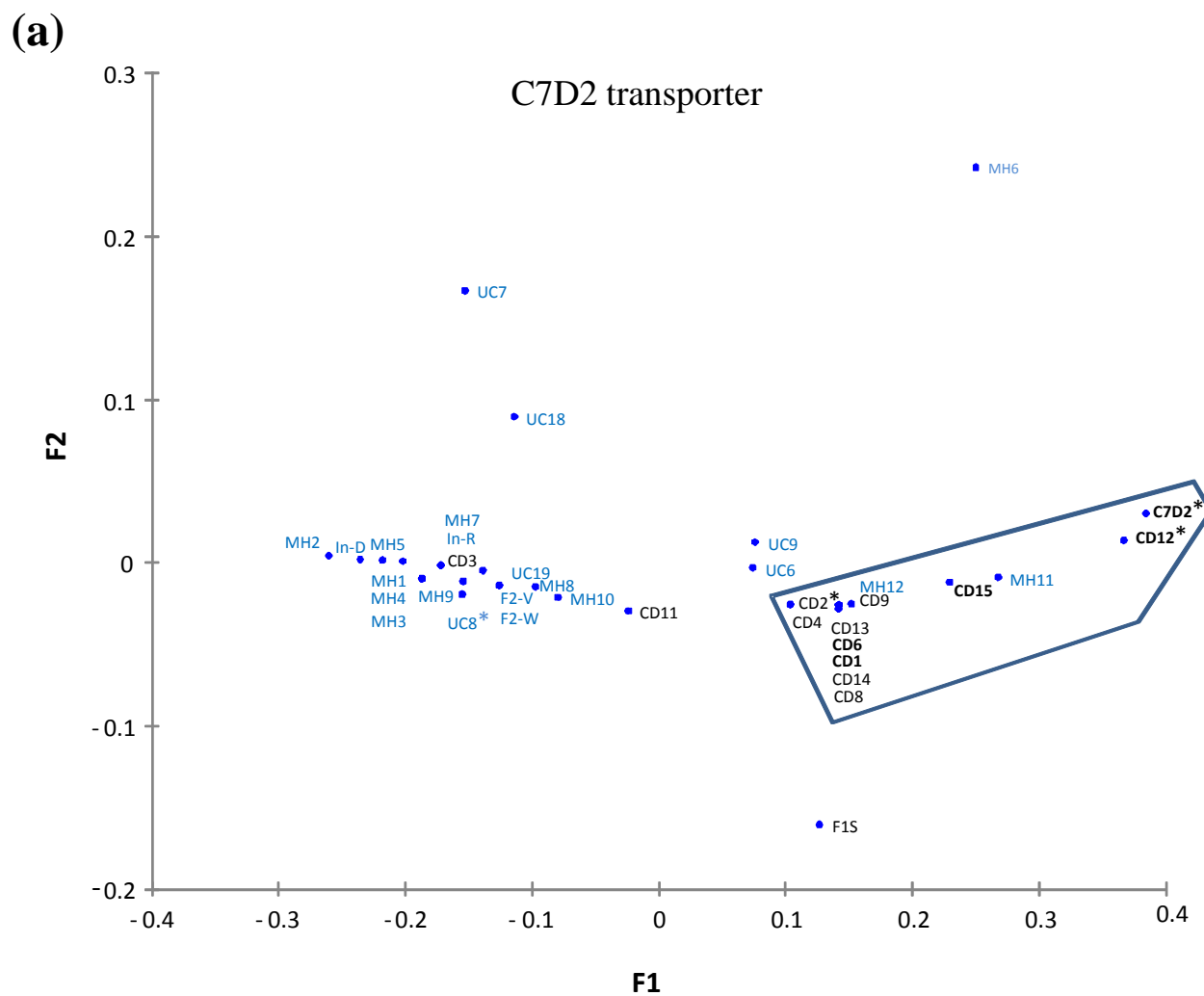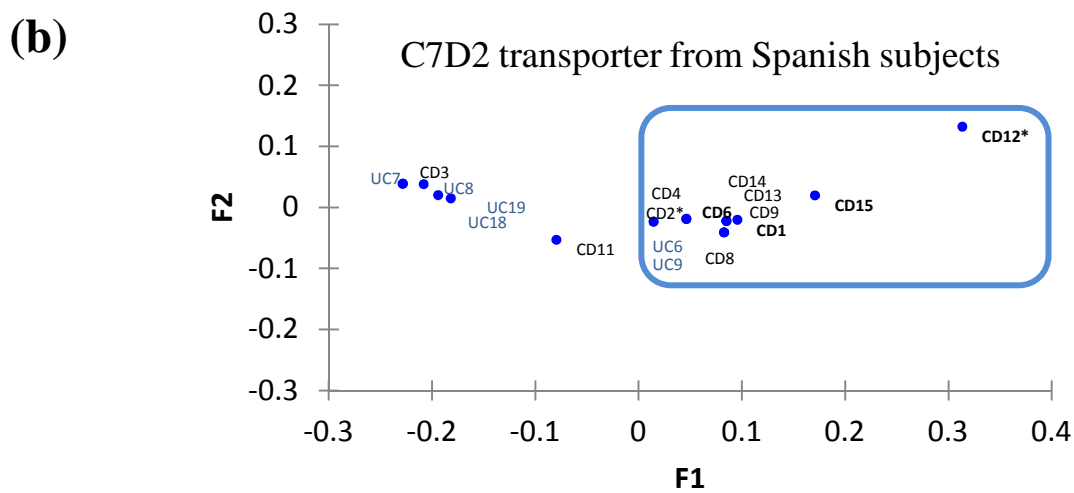

**S3 Figure**

Supplement: S3 Fig — Principal coordinate analyses (PCoA) ordination plots, based on Bray-Curtis distances were performed with identity percentages recovered from the cohort members using metagenomic C7D2 transporter as query. (a) All CDR and CDU tested. (b) Only Spanish subjects. *: truncated protein, blue: CDU subjects, black: CDR asymptomatic subjects, bold black: CD patients. (PDF) [file pone.0148291.s003.pdf]

GUS  $\beta$ -glucuronidases

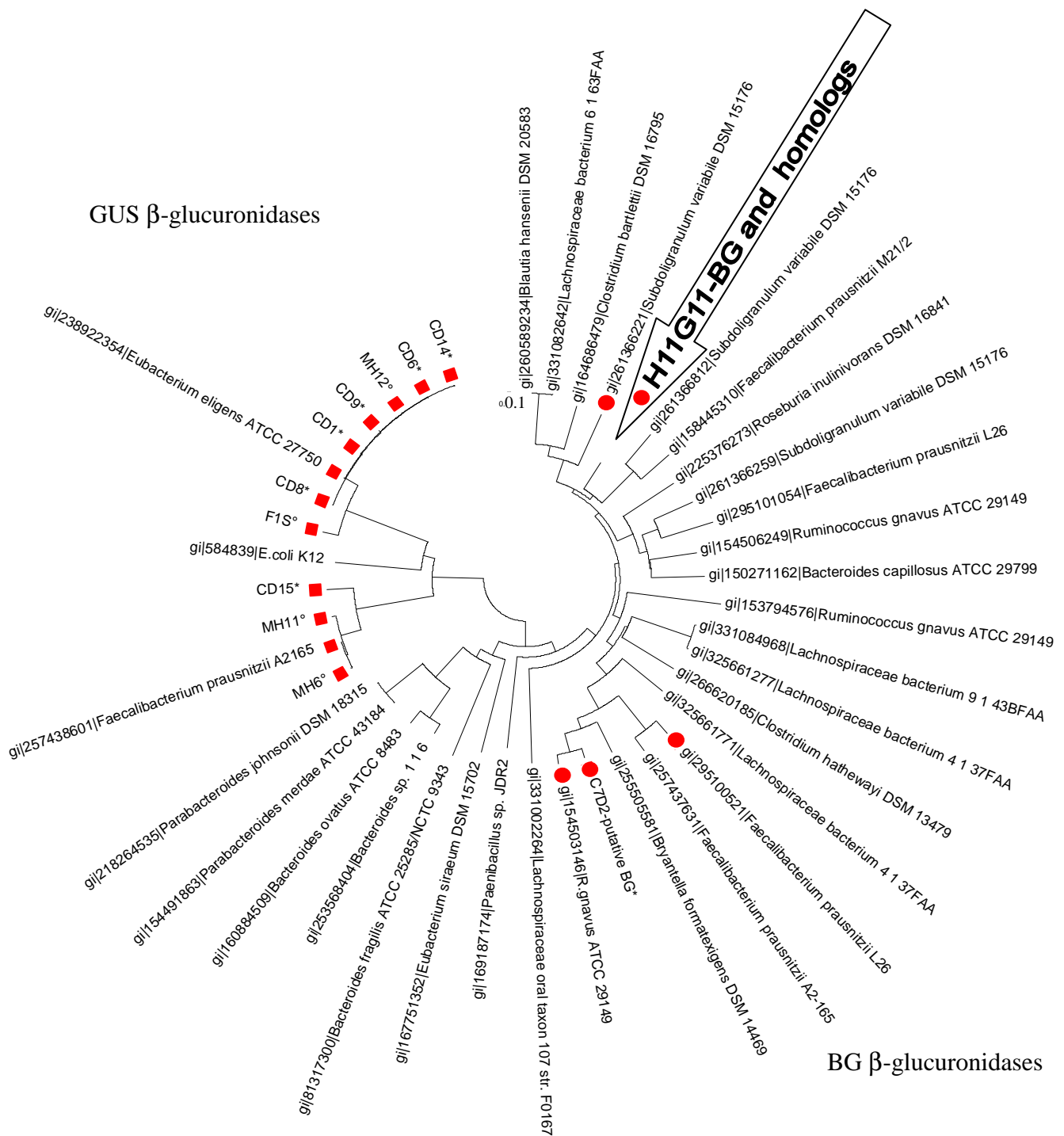

S4 Figure

Supplement: S4 Fig — The Neighbor-Joining tree was performed using MEGA 5.5 on β-glucuronidase sequences close to C7D2-and H11G11- transporters from microbiomes and to C7D2-transporters from published genomes. Red squares and circles represent respectively GUS and BG β-glucuronidases neighboring a C7D2-transporter. Δ metagenomic clone from CD patient (E. coli as the host), * microbiome from CDR including patients and unaffected first degree relatives, ° microbiome from healthy subject. (PDF) [file pone.0148291.s004.pdf]

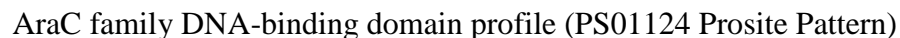

## S5 Figure

Supplement: S5 Fig — The AraC-like sequences retrieved from C7D2 transporter loci (mostly from CDR microbiomes) and published genomes were aligned using the Tcoffee expresso tool (TCoffee score 96–98). The COOH-terminal domain encodes the DNA binding domain. (PDF) [file pone.0148291.s005.pdf]

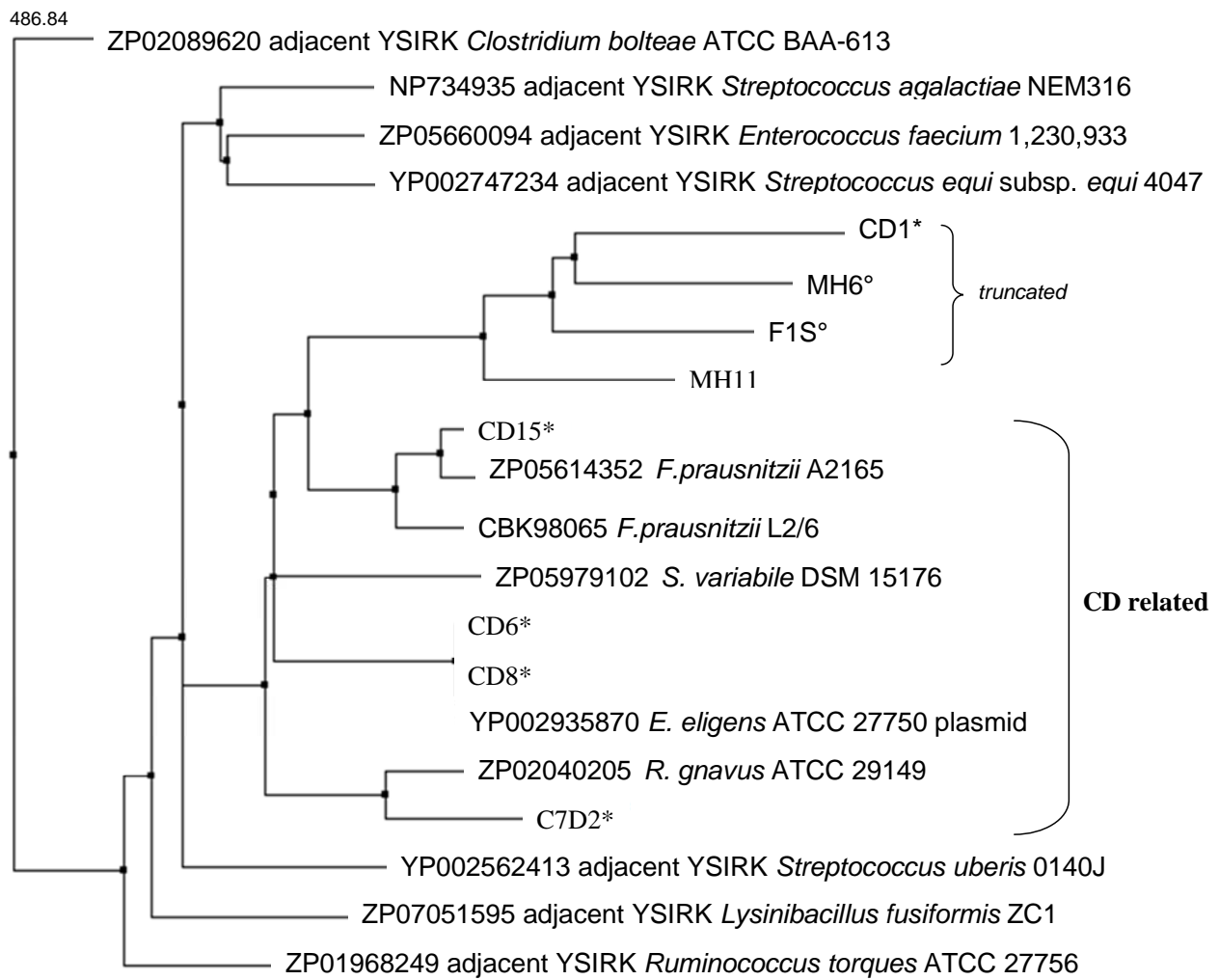

**S6 Figure**

Supplement: S6 Fig — Neighbor-Joining tree was performed using BLOSUM62 on region after ClustalW alignment from AFTR sequences co-localized with groups 1 and 2 C7D2-transporters (see Fig 1) and those of the best homologs (YSIRK-targeted surface antigen AFTR) found in published genomes. *: CDR, °: healthy subject. AFTRs grouped like their respective co-localized C7D2 transporters. (PDF) [file pone.0148291.s006.pdf]

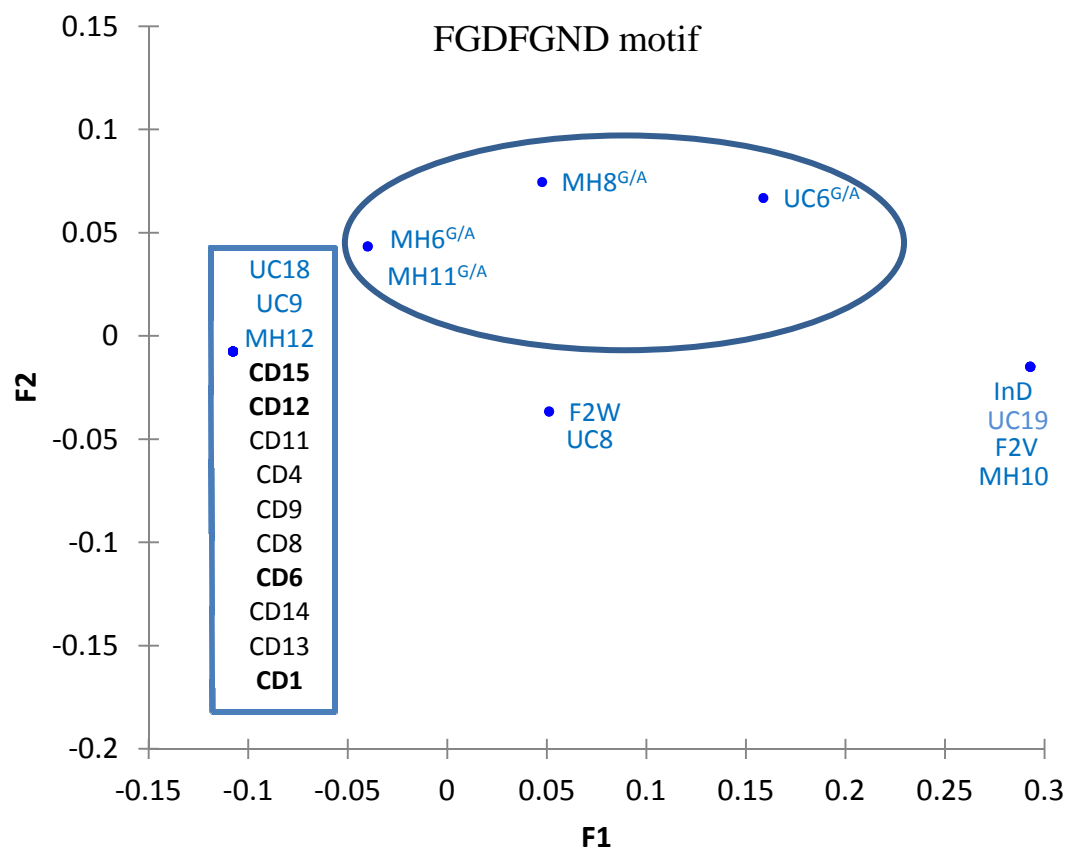

S8 Figure

Supplement: S8 Fig — Principal coordinate analyses (PCoA) ordination plots, based on Bray-Curtis distances were performed with identity percentages recovered from the cohort members using the FGDFGND motif from the metagenomic C7D2 transporter as query. CDU subjects, black: CDR asymptomatic subjects, bold black: CD patients. (PDF) [file pone.0148291.s008.pdf]
