# Supplementary material for: Unique β-Glucuronidase Locus in Gut Microbiomes of Crohn’s Disease Patients and Unaffected First-Degree Relatives
Source: PLoS One. 2016 Jan 29;11(1):e0148291. doi: 10.1371/journal.pone.0148291 (PMC4732671; doi:10.1371/journal.pone.0148291)
Supplement: S7 Fig — The Alignment was performed using Expresso multiple alignment tool of protein sequences using structural information (T-COFFEE Multiple Sequence Alignment Server). (PDF) [file pone.0148291.s007.pdf]

```
T-COFFEE, Version_11.00.8cbe486 (2014-08-12 22:05:29 - Revision 8cbe486 - Build 477)
Cedric Notredame
CPU TIME:0 sec.
SCORE=97
*
 BAD AVG GOOD
*
C7D2 CD1      :  98
C7D2 CD4      :  96
C7D2 CD6      :  98
C7D2 CD8      :  98
C7D2 CD9      :  98
C7D2 CD11     :  96
C7D2 CD12     :  97
C7D2 CD13     :  98
C7D2 CD14     :  98
C7D2 CD15     :  97
cons          :   9

C7D2_CD1      MT---DNNGKVRPFGIKDKLGYMFGDFGNDFTFLLSAMFLLKFYTDVMGVSAALVGLMMMAARFVDAIT
C7D2_CD4      MK----NDKIVKPFGFKDKIGYMFGDFGNDLTFLLSSSFLMKFYTDVMDVDGYIVGIIMMIARFIDAFT
C7D2_CD6      MT---DNNGKVRPFGIKDKLGYMFGDFGNDFTFLLSAMFLLKFYTDVMGVSAALVGLMMMAARFVDAIT
C7D2_CD8      MT---DNNGKVRPFGIKDKLGYMFGDFGNDFTFLLSAMFLLKFYTDVMGVSAALVGLMMMAARFVDAIT
C7D2_CD9      -------NGKVRPFGIKDKLGYMFGDFGNDFTFLLSAMFLLKFYTDVMGVSAALVGLMMMAARFVDAIT
C7D2_CD11     ME----TNKTIRPFGMRDKIGYLFGDFGNDVMLIFVSQFLMVFYTQVWGMKPAVVGTMFLVSRLVDAFT
C7D2_CD12     MKMGKTNNSTVPAFGMKDRLGYMFGDFGNDFTFILSSMFMMKFYTDVMGLSAGVVGMLMMAARFVDAFT
C7D2_CD13     MT---DNNGKVRPFGIKDKLGYMFGDFGNDFTFLLSAMFLLKFYTDVMGVSAALVGLMMMAARFVDAIT
C7D2_CD14     MT---DNNGKVRPFGIKDKLGYMFGDFGNDFTFLLSAMFLLKFYTDVMGVSAALVGLMMMAARFVDAIT
C7D2_CD15     MA---NEMKKAAPFGMKDKVGYMFGDFGNDFTFLLSSSFLMKFYTDVMGVNAAIVGMVMMIARFVDAFT

cons                   .**::*::**:*******. ::: : *:: ***:* .:.  :** ::: :*::**:*


C7D2_CD1      DVTMGQIVDRSRPGKKGKFAPWLRRMCGPVALASFLMYATWFKDMPMGFKIFWMFFTYLLWGSICYTGI
C7D2_CD4      DVAMGRICDKSKMTAAGKFKPWIRRMCGPVAIVSFLMYQSSLSGLPKPAKIAYLFVTYILWGSVFYTSI
C7D2_CD6      DVTMGQIVDRSRPGKKGKFAPWLRRMCGPVALASFLMYATWFKDMPMGFKIFWMFFTYLLWGSICYTGI
C7D2_CD8      DVTMGQIVDRSRPGKKGKFAPWLRRMCGPVALASFLMYATWFKDMPMGFKIFWMFFTYLLWGSICYTGI
C7D2_CD9      DVTMGQIVDRSRPGKKGKFAPWLRRMCGPVALASFLMYATWFKDMPMGFKIFWMFFTYLLWGSICYTGI
C7D2_CD11     DITMGRIVDTAPQGKDGKFKKWIRIMAAPVAIASFLMYQSFLRDYPMIVKIVYMYITYLLYGSICFTGI
C7D2_CD12     DVTMGQIVDRSKPTKDGKFRPWIKRMCGPVAIASFLIYQSGLADMPYGVKVAYMVVTYLLWGSIFYTSI
C7D2_CD13     DVTMGQIVDRSRPGKKGKFAPWLRRMCGPVALASFLMYATWFKDMPMGFKIFWMFFTYLLWGSICYTGI
C7D2_CD14     DVTMGQIVDRSRPGKKGKFAPWLRRMCGPVALASFLMYATWFKDMPMGFKIFWMFFTYLLWGSICYTGI
C7D2_CD15     DVGMGQIVDHSKPNKDGKFRPWIKRMCGPVAIASFLIYQSGLAGMPYGFKVAWLFVTYILWGSIFYTSI

cons          *: **:* * :      ***  *:: *..***:.***:* : : . *   *: :: .**:*:**: :*.*


C7D2_CD1      NIPYGSMASAISDNPTDRTSLSNWRTIGATLAQTAIGVVLPLVVYYT---DEAGNSVL--SGQKMMLGA
C7D2_CD4      NIPYGSMASAISDNPNDRQSLSTFRTMGGMFAGAIIMAVVPLIIYNK----ENGNDVI--SGAKFTVVA
C7D2_CD6      NIPYGSMASAISDNPTDRTSLSNWRTIGATLAQTAIGVVLPLVVYYT---DEAGNSVL--SGQKMMLGA
C7D2_CD8      NIPYGSMASAISDNPTDRTSLSNWRTIGATLAQTAIGVVLPLVVYYT---DEAGNSVL--SGQKMMLGA
C7D2_CD9      NIPYGSMASAISDNPTDRTSLSNWRTIGATLAQTAIGVVLPLVVYYT---DEAGNSVL--SGQKMMLGA
C7D2_CD11     NIPYGAMASAITDKPDERQSLITFRAMGAYIGEFLIGFFGPLVIYQRIITDEGVQVVIRNNGNIFPVAA
C7D2_CD12     NIPYGSMASAISPDPTDRASLSTWRSVGASLASLVIGTGTPLVAYVT----VDGNPVL--SGSRMTIIA
C7D2_CD13     NIPYGSMASAISDNPTDRTSLSNWRTIGATLAQTAIGVVLPLVVYYT---DEAGNSVL--SGQKMMLGA
C7D2_CD14     NIPYGSMASAISDNPTDRTSLSNWRTIGATLAQTAIGVVLPLVVYYT---DEAGNSVL--SGQKMMLGA
C7D2_CD15     NIPYGSMASAISSDPKDRADLSTWRTIGSTLANMVIGVATPMVAYVV----VDGKTVM--SGSRMTIIA

cons          *****:*****: .* :* .* .:*::*. :.   *   *:: *         : *:  .*  : : *


C7D2_CD1      LVCSIGAVICYMLCYKMTTERVKVGQN---T-QKFSFGELIKELAHNRSLIGIIVCALVFLLAQLSLSN
C7D2_CD4      GVCSLLAVVCYLLCYALTTERVRAQATDAQL-EKNNLGVMLKNAVKNRALISIIVASIFMLISQLTIQQ
C7D2_CD6      LVCSIGAVICYMLCYKMTTERVKVGQN---T-QKFSFGELIKELAHNRSLIGIIVCALVFLLAQLSLSN
C7D2_CD8      LVCSIGAVICYMLCYKMTTERVKVGQN---T-QKFSFGELIKELAHNRSLIGIIVCALVFLLAQLSLSN
C7D2_CD9      LVCSIGAVICYMLCYKMTTERVKVGQN---T-QKFSFGELIKELAHNRSLIGIIVCALVFLLAQLSLSN
C7D2_CD11     GGISLIAIVCYYICYTCTTERIKVPAL---SKEGVSFASSVKMIFSNRAMIGILGGTVCLIFGNLLTGG
C7D2_CD12     GVFSVLAVICYLLCYNLVRERVPVPAN---N-QKLDIVALGKSLITNRALLGIIAAALLLLAMLGMQG
C7D2_CD13     LVCSIGAVICYMLCYKMTTERVKVGQN---T-QKFSFGELIKELAHNRSLIGIIVCALVFLLAQLSLSN
C7D2_CD14     LVCSIGAVICYMLCYKMTTERVKVGQN---T-QKFSFGELIKELAHNRSLIGIIVCALVFLLAQLSLSN
C7D2_CD15     GVFSICAILCYIVCFKLTSERVEVPAS---N-QKISASAIFKSIFTNKALLGIIVAAIFVLLSQLTVLS

cons             *: *::** :*:  . **: .       :  .     *    *::::.*:  :: .::. *


C7D2_CD1      MNAYIYPNYFGNIKAMSIA--SLSGTIVIL-LLSTFITKLASKIGKKELSVIGCIISAASFITLFIIHT
C7D2_CD4      MANYVFPNYYGNAKVQSLSVVMMGGGM---VIAAVIAKPLAAKFGKAEISVVSNMVAGVVSLLLYFVRP
C7D2_CD6      MNAYIYPNYFGNIKAMSIA--SLSGTIVIL-LLSTFITKLASKIGKKELSVIGCIISAASFITLFIIHT
C7D2_CD8      MNAYIYPNYFGNIKAMSIA--SLSGTIVIL-LLSTFITKLASKIGKKELSVIGCIISAASFITLFIIHT
C7D2_CD9      MNAYIYPNYFGNIKAMSIA--SLSGTIVIL-LLSTFITKLASKIGKKELSVIGCIISAASFITLFIIHT
C7D2_CD11     VNAYLYAYYFKMPEALSTY--NAIKLGVAL-AMATGVTLVVKKIGRRESISVMAGLASLVFVLLFFLRI
C7D2_CD12     MSAYVFPNVYRSTQAQSVV--ALLSNVAIIAICAPLASKLASKFGKKELATVSCLFGAASYVVCLILHP
C7D2_CD13     MNAYIYPNYFGNIKAMSIA--SLSGTIVIL-LLSTFITKLASKIGKKELSVIGCIISAASFITLFIIHT
C7D2_CD14     MNAYIYPNYFGNIKAMSIA--SLSGTIVIL-LLSTFITKLASKIGKKELSVIGCIISAASFITLFIIHT
C7D2_CD15     LAGYVYPNVYGSAAAQSTA--SLLGTVVMLVVCAPFASKLSAKFGKKELAMGSSIFSALVWLVCLIWRP

cons          :  *::.  :     . *             :   . :  *:*: *         ...    :    : :


C7D2_CD1      HNVWIWIALIIIATIGTSMFNMVIWAMITDVIDESEVQNGVRQDGTIYSVYSFARKLGQACSSGLAGVL
C7D2_CD4      QNVWVYVALQFLCWFGLGVFSMVVWALITDVIDYSEIKNGIREDGSVYALYSFARKLGQALTSGLSGAL
C7D2_CD6      HNVWIWIALIIIATIGTSMFNMVIWAMITDVIDESEVQNGVRQDGTIYSVYSFARKLGQACSSGLAGVL
C7D2_CD8      HNVWIWIALIIIATIGTSMFNMVIWAMITDVIDESEVQNGVRQDGTIYSVYSFARKLGQACSSGLAGVL
C7D2_CD9      HNVWIWIALIIIATIGTSMFNMVIWAMITDVIDESEVQNGVRQDGTIYSVYSFARKLGQACSSGLAGVL
C7D2_CD11     KNPWVYVAISSVGFSGVTFFGYTVWGAIIDVIDDAEIKSGKREDGTLYAIYSFSRKIGQALGSSIVGYA
C7D2_CD12     ENVWVYVIFYVMSFVGLGFFNTIIWAMITDVIDDAEVKNGIREDGTIYAVYSFARKLGQAFSSGMVGGL
C7D2_CD13     HNVWIWIALIIIATIGTSMFNMVIWAMITDVIDESEVQNGVRQDGTIYSVYSFARKLGQACSSGLAGVL
C7D2_CD14     HNVWIWIALIIIATIGTSMFNMVIWAMITDVIDESEVQNGVRQDGTIYSVYSFARKLGQACSSGLAGVL
C7D2_CD15     ASVWGFVACYILANIGMGFFNTIIWAMITDVIDDAEVRNGVREDGTIYSVYSFARKLGQAFSSGLVGAL

cons           . * ::     :    *  .*.   :*. * **** :*::.* *:**::*::***:**:***  *.: *


C7D2_CD1      LSIVGYTTATAF-DPKVIDGIYNVTCLMPAAGMTLLLLALIFLYPLNKKRVEANAAKLREIRAAKEQQ
C7D2_CD4      LSMIGYKKSTAF-ESQVKEGIFDISTLVPAISFILLALILWFWYPLKKKLVDENVEFLRKKHNKTE-E
C7D2_CD6      LSIVGYTTATAF-DPKVIDGIYNVTCLMPAAGMTLLLLALIFLYPLNKKRVEANAAKLREIRAAKEQQ
C7D2_CD8      LSIVGYTTATAF-DPKVIDGIYNVTCLMPAAGMTLLLLALIFLYPLNKKRVEANAAKLREIRAAKEQQ
C7D2_CD9      LSIVGYTTATAF-DPKVIDGIYNVTCLMPAAGMTLLLLALIFLYPLNKKRVEANAAKLREIRAAKEQQ
C7D2_CD11     [illegible]
```

S7 Figure
