# Supplementary material for: Unique β-Glucuronidase Locus in Gut Microbiomes of Crohn’s Disease Patients and Unaffected First-Degree Relatives
Source: PLoS One. 2016 Jan 29;11(1):e0148291. doi: 10.1371/journal.pone.0148291 (PMC4732671; doi:10.1371/journal.pone.0148291)
Supplement: S1 Table — (PDF) [file pone.0148291.s009.pdf]

S1 Table. Summary of the samples and sequencing.

Sample name	Disease status	Country	Sex	Age (years)	BMI	Total length of assembled sequence (Mb)	References
In-A	healthy	Japan	Male	45		29.93	
In-D	healthy	Japan	Male	35		49.55	
In-R	healthy	Japan	Female	24		46.79	
F1-S	healthy	Japan	Male	30		38.86	(Kurokawa, Itoh et al. 2007)[1]
F1-T	healthy	Japan	Female	28		44.28	
F2-V	healthy	Japan	Male	37		47.02	
F2-W	healthy	Japan	Female	36		40.97	
MH0001	healthy	Denmark	Female	49	25.55	19.69	
MH0002	healthy	Denmark	Female	59	27.28	88.77	
MH0003	healthy	Denmark	Male	69	33.19	119.59	
MH0004	healthy	Denmark	Male	59	31.18	31.92	
MH0005	healthy	Denmark	Male	69	21.68	19.62	
MH0006	healthy	Denmark	Female	59	22.38	217.77	
MH0007	healthy	Denmark	Male	69	33.60	32.00	
MH0008	healthy	Denmark	Male	59	24.35	37.24	
MH0009	healthy	Denmark	Male	64	29.04	112.96	
MH0010	healthy	Denmark	Male	64	33.27	36.52	
MH0011	healthy	Denmark	Female	0	22.31	134.25	
MH0012	healthy	Denmark	Female	42	32.10	237.58	
CD1	patient	Spain	Female	25	17.93	54.83	
CD2	first–degree relative	Spain	Male	49	27.76	12.26	
CD3	first–degree relative	Spain	Female	18	21.51	74.88	(Qin, Li et al. 2010)[2]
CD4	first–degree relative	Spain	Female	46	29.69	100.99	
CD6	patient	Spain	Female	36	18.52	76.79	
CD8	first–degree relative	Spain	Male	51	29.38	116.87	
CD9	first–degree relative	Spain	Female	48	27.55	99.57	
CD11	first–degree relative	Spain	Female	62	35.46	83.58	
CD12	patient	Spain	Female	41	20.20	59.20	
CD13	first–degree relative	Spain	Male	68	25.69	99.94	
CD14	first–degree relative	Spain	Female	41	23.12	116.12	
CD15	patient	Spain	Female	34	19.00	56.10	
UC6	healthy	Spain	Female	38	23.18	125.25	
UC7	healthy	Spain	Female	19	23.05	56.97	
UC8	healthy	Spain	Male	22	25.40	121.81	
UC9	healthy	Spain	Male	32	30.37	99.85	
UC18	healthy	Spain	Female	63	28.67	94.28	
UC19	healthy	Spain	Female	37	21.19	113.78	
