# Supplementary material for: Unique β-Glucuronidase Locus in Gut Microbiomes of Crohn’s Disease Patients and Unaffected First-Degree Relatives
Source: PLoS One. 2016 Jan 29;11(1):e0148291. doi: 10.1371/journal.pone.0148291 (PMC4732671; doi:10.1371/journal.pone.0148291)
Supplement: S2 Table — (PDF) [file pone.0148291.s010.pdf]

S2 Table. Best tBlastn results for H11G11- and C7D2- transporters in microbiomes of CD patients, first degree relatives and healthy subjects.

Microbiome source	Database disease status	Template H11G11 (healthy) C7D2 (CD)	C7D2 (CD) homology (identity%)	H11G11 (Healthy) homology (identity%)	Best Blastp result with identified strain (identity%)	Name given (template-microbiome)
F1-S	Healthy	H11G11	27	**33**	*gi160943280 Faecalibacterium prausnitzii M21/2 (99%)*	H11G11 F1S
		C7D2	**52**	30	*gi238874027 Eubacterium eligens ATCC 27750 plasmid (90%)*	C7D2 F1S
F2-V	Healthy	C7D2	30	29	*gi182624781 Clostridium perfringens D str. JGS1721 (42%)*	C7D2 F2V
F2-W	Healthy	H11G11/C7D2	30	**100**	*gi154505099 Ruminococcus gnavus ATCC 29149 (44%)*	H11G11/C7D2 F2W
In-D	Healthy	H11G11 /C7D2	23	23	*gi189459801 Bacteroides coprocola DSM 17136 (99%)*	C7D2/H11G11 InD
In-R	Healthy	H11G11	27	**34**	*gi 291534767 Roseburia intestinalis M50/1 (99%)*	H11G11 InR
MH0001	Healthy	H11G11 /C7D2	25	25	*gi153813566 Ruminococcus obeum ATCC 29174 (73%)*	H11G11/C7D2 MH1
MH0002	Healthy	H11G11	21	25	*gi269959236 Vibrio harveyi 1DA3 (31%)*	H11G11 MH2
MH0003	Healthy	H11G11	26	**87**	*gi154505099 Ruminococcus gnavus ATCC 29149 (44%)*	H11G11 MH3
MH0004	Healthy	H11G11	26	27	*gi291524604 Eubacterium rectale DSM 17629 (100%)*	H11G11 MH4
MH0005	Healthy	H11G11/ C7D2	24	25	*gi153813566 Ruminococcus obeum ATCC 29174 (72%)*	H11G11/C7D2 MH5
MH0006	Healthy	H11G11	26	**87**	*gi154505099 Ruminococcus gnavus ATCC 29149 (44%)*	H11G11 MH6
		C7D2	**70**	29	*gi257438598 Faecalibacterium prausnitzii A2-165 (99%)*	C7D2 MH6
MH0007	Healthy	H11G11 /C7D2	27	33	*gi 291534767 Roseburia intestinalis M50/1 (99%)*	C7D2/ H11G11 MH7
MH0008	Healthy	H11G11 /C7D2	32	**46**	*gi260589235 Blautia hansenii DSM 20583 (58%)*	H11G11 /C7D2 MH8
MH0009	Healthy	H11G11 /C7D2	28	**100**	*gi154505099 Ruminococcus gnavus ATCC 29149 (45%)*	H11G11/C7D2 MH9
MH0010	Healthy	C7D2	**33**	29	*gi257413542 Roseburia intestinalis L1-82 (100%)*	C7D2/H11G11 MH10
MH0011	Healthy	C7D2	**69**	29	*gi257438598 Faecalibacterium prausnitzii A2-165 (99%)*	C7D2 MH11
MH0012	Healthy	H11G11	28	**100**	*gi154505099 Ruminococcus gnavus ATCC 29149 (44%)*	H11G11 MH12
		C7D2	**52**	29	*gi238874027 Eubacterium eligens ATCC 27750 plasmid (99%)*	C7D2 MH12
V1-UC6	Healthy	H11G11/ C7D2	**45**	30	*gi347533214 Roseburia hominis A2-183 (100%)*	C7D2 /H11G11 UC6
V1-UC7	Healthy	H11G11/ C7D2	27	29	*gi494990662 Clostridium asparagiforme (85%)*	H11G11/ C7D2 UC7
V1-UC8	Healthy	H11G11/ C7D2	28	**99**	*gi547236978 Eubacterium sp. CAG:180 (99%)*	H11G11/ C7D2 UC8
V1-UC9	Healthy	H11G11/ C7D2	**45**	30	*gi548310806 Roseburia hominis A2-183 (58%)*	C7D2 /H11G11 UC9
V1-UC18	Healthy	H11G11	27	26	*gi547465828 Roseburia sp. CAG:197 (95%)*	H11G11 UC18<br>C7D2
		C7D2	30	22	*gi479208413 Faecalibacterium prausnitzii L2-6 (98%)*	C7D2 UC 18
V1-UC19	Healthy	H11G11/ C7D2	29	28	*gi479149068 Roseburia intestinalis XB6B4 (100%)*	C7D2/H11G11 UC19
V1-CD13	CD-FR	C7D2	**52**	29	*gi238874027 Eubacterium eligens ATCC 27750 plasmid (99%)*	C7D2 CD13
V1.CD-2	CD-FR	C7D2	**48***	34*		
V1.CD-3	CD-FR	H11G11	27	**36**	*gi154505099 Ruminococcus gnavus ATCC 29149 (42%)*	H11G11 CD3
V1.CD-4	CD-FR	C7D2	**48**	33	*gi261366220 Subdoligranulum variabile DSM 15176 (66%)*	C7D2/H11G11 CD4
V1.CD-8	CD-FR	H11G11	26	**87**	*gi154505099 Ruminococcus gnavus ATCC 29149 (44%)*	H11G11 CD8
		C7D2	**52**	30	*gi238874027 Eubacterium eligens ATCC 27750 plasmid (99%)*	C7D2 CD8
V1.CD-9	CD-FR	C7D2	**53**	30	*gi238874027 Eubacterium eligens ATCC 27750 plasmid (99%)*	C7D2 CD9
V1.CD-11	CD-FR	C7D2	**37**	29	*gi237737733 Fusobacterium mortiferum ATCC 9817 (100%)*	C7D2 CD11
V1.CD-14	CD-FR	C7D2	**52**	30	*gi238874027 Eubacterium eligens ATCC 27750 plasmid (99%)*	C7D2 CD14
V1.CD-1	CD	H11G11	27	**33**	*gi160943280 Faecalibacterium prausnitzii M21/2 (99%)*	H11G11 CD1
		C7D2	**52**	29	*gi238874027 Eubacterium eligens ATCC 27750 plasmid (99%)*	C7D2 CD1
V1.CD-6	CD	C7D2	**52**	30	*gi238874027 Eubacterium eligens ATCC 27750 plasmid (99%)*	C7D2 CD6
V1.CD-12	CD	H11G11	28	31	*gi 291534591 Roseburia intestinalis M50/1 (80%)*	H11G11 CD12
		C7D2	**92**	31	*gi154503147 Ruminococcus gnavus ATCC 29149 (83%)*	C7D2 CD12
V1.CD-15	CD	C7D2	**63**	31	*gi257438598 Faecalibacterium prausnitzii A2-165 (75%)*	C7D2 CD15
**Template H11G11-transporter**			**29**	**100**	*gi154505099 Ruminococcus gnavus ATCC 29149 (44%)*	
**Template C7D2-transporter**			**100**	**29**	*gi154505099 Ruminococcus gnavus ATCC 29149 (83%)*	

Spanish subjects are underlined. They include both CD-related families and healthy subjects (CD-unrelated). *: truncated sequences (<150 amino acids).
