# Supplementary material for: Unique β-Glucuronidase Locus in Gut Microbiomes of Crohn’s Disease Patients and Unaffected First-Degree Relatives
Source: PLoS One. 2016 Jan 29;11(1):e0148291. doi: 10.1371/journal.pone.0148291 (PMC4732671; doi:10.1371/journal.pone.0148291)
Supplement: S3 Table — (PDF) [file pone.0148291.s011.pdf]

S3 Table. Coverage and identity of amino acid AFTR sequences associated to C7D2-transporter in published genomes

Strains	homology with AFTR from *E. eligens* plasmid	
	Coverage (%)	Identity (%)*
Clostridium hathewayi DSM 13479	99	37
Faecalibacterium prausnitzii L2-6	96	30
Faecalibacterium prausnitzii A2-165	96	31
Ruminococcus gnavus ATCC 29149	99	40
Shuttleworthia satelles DSM 14600	98	40
Subdoligranulum variabile DSM 15176	84	35
Roseburia hominis A2-183	83	38
Carnobacterium sp. AT7	80	32
Clostridium bartlettii DSM 16795	76	31
Roseburia intestinalis L1-82	58	25
Clostridium asparagiforme DSM 15981	57	33
Clostridium cellulolyticum H10	52	37

*: determined from homologous region from the blastp result.
